# Acquisition‐Recruited and MOR‐Expressing Striatal dMSNs Differentially Regulate Extinction and Cue‐Induced Relapse of Alcohol Seeking

**DOI:** 10.1111/adb.70191

**Published:** 2026-09-13

**Authors:** Xueyi Xie, Xuehua Wang, Jun Wang

**Affiliations:** ^1^ Department of Neuroscience and Experimental Therapeutics Naresh K. Vashisht College of Medicine, Texas A&M University Health Science Center Bryan Texas USA

**Keywords:** alcohol use disorder, ArcTRAP/FosTRAP, direct‐pathway medium spiny neurons, dorsomedial striatum, engram ensemble, extinction, operant self‐administration, relapse, striosome

## Abstract

Relapse to alcohol use is frequently triggered by re‐exposure to alcohol‐associated cues, and extinction‐based interventions can reduce this vulnerability. However, the striatal cellular mechanisms through which extinction alters later alcohol seeking remain unclear. Using a mouse model of operant alcohol self‐administration, we examined two distinct direct‐pathway medium spiny neuron (dMSN) populations in the dorsomedial striatum. Extinction reduced cue‐induced alcohol seeking and decreased the probability that acquisition‐recruited dMSNs were reactivated during relapse testing. Chemogenetically maintaining activity in acquisition‐recruited dMSNs during extinction slowed the reduction in alcohol‐seeking and enhanced subsequent relapse. In contrast, increasing activity in μ‐opioid receptor (MOR)‐expressing dMSNs, a striosome‐enriched population, accelerated the reduction in responding and decreased later relapse without altering inactive‐lever responding or locomotor activity. These findings indicate that extinction is associated with reduced re‐engagement of acquisition‐related dMSN ensembles, whereas enhancing activity in a MOR‐defined, striosome‐enriched output population can facilitate extinction‐related behavioural change. Together, the results identify two complementary striatal processes that regulate alcohol extinction and relapse.

## Graphical abstract

Extinction training reduced cue‐induced alcohol seeking and reactivation of acquisition‐recruited dorsomedial striatal dMSN ensembles. Maintaining acquisition‐ensemble activity during extinction increased later relapse, whereas activating MOR‐expressing, striosome‐enriched dMSNs facilitated extinction‐related behavioural change and reduced subsequent alcohol seeking, revealing complementary striatal mechanisms that regulate extinction and relapse.

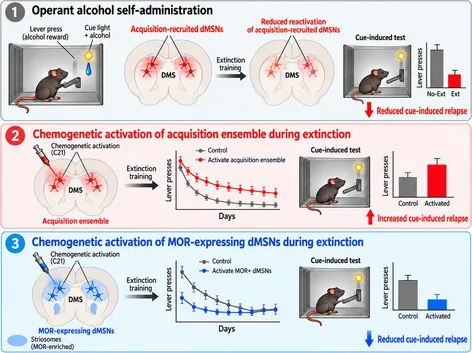

## 1 Introduction

Relapse remains one of the greatest challenges in the treatment of alcohol and other substance use disorders [1, 2, 3]. Even after prolonged abstinence, exposure to drug‐associated cues, contexts or stress can trigger renewed drug seeking, reflecting the persistence of maladaptive drug‐related memories [1, 2, 4]. Extinction‐based interventions can reduce relapse vulnerability in both clinical and preclinical settings [5, 6, 7, 8, 9, 10], but extinction does not erase the original association. Instead, extinction establishes new learning that suppresses expression of the original memory, which can re‐emerge when conditions change [9, 11, 12, 13, 14]. Identifying the cellular mechanisms that support this suppression is therefore important for improving extinction‐based approaches. In particular, studies of reward‐omission and anti‐relapse neuronal ensembles suggest that defined neuronal populations can actively constrain drug seeking [15, 16].

The reduced expression of an acquired drug memory and the formation of an extinction memory should therefore be viewed as complementary rather than competing processes. During extinction, animals learn that a previously reinforced action or cue no longer produces the expected outcome. The resulting extinction memory can inhibit behavioural expression of the original association without eliminating its underlying representation [9, 10, 11, 17]. How this behavioural updating changes the recruitment and subsequent accessibility of drug‐related neuronal ensembles remains an important question in addiction neuroscience.

The dorsomedial striatum (DMS) plays a critical role in goal‐directed action and alcohol seeking [5, 18, 19, 20, 21, 22, 23]. Within the striatum, direct‐pathway medium spiny neurons (dMSNs) promote action execution and instrumental reward learning [24, 25, 26, 27, 28, 29]. Across addiction models, sparse activity‐defined neuronal ensembles in the medial prefrontal cortex, nucleus accumbens, infralimbic cortex and DMS have been shown to encode drug‐associated contexts or actions and causally regulate subsequent drug seeking [30, 31, 32, 33]. Recent evidence further indicates that subsets of DMS dMSNs recruited during acquisition of operant alcohol self‐administration form ensembles that encode alcohol‐associated memories [34, 35, 36, 37, 38]. Reactivation of these acquisition‐recruited dMSN ensembles during cue exposure drives relapse‐like behaviour, suggesting that persistent striatal activity patterns contribute directly to relapse of alcohol seeking.

One possibility is that extinction suppresses subsequent alcohol seeking by reducing reactivation of these acquisition‐recruited dMSNs. This model is consistent with studies showing that reward‐omission and extinction‐responsive ensembles can oppose reward‐ or drug‐seeking populations and suppress subsequent responding [15, 16, 39, 40]. However, whether extinction reduces later re‐engagement of acquisition‐recruited DMS dMSNs has not been directly examined. It also remains unclear whether maintaining activity in these neurons during extinction alters extinction‐related behaviour and later relapse.

Extinction also requires updating the expected consequences of a previously reinforced action [9, 11, 41]. The striatum contains matrix and striosome compartments that differ in connectivity, molecular identity and function [42, 43, 44, 45]. Striosomes constitute approximately 10%–15% of the striatum, depending on species and subregion, whereas the surrounding matrix comprises the majority [46, 47, 48]. Despite their relatively limited representation, emerging studies suggest that, in contrast to matrix dMSNs, which are broadly involved in positive reinforcement and behavioural execution, striosomal dMSNs may be preferentially involved in negative reinforcement and behavioural inhibition [42, 45, 49, 50, 51, 52]. Our recent work further showed that dMSNs recruited during extinction are preferentially enriched in striosomes, whereas acquisition‐recruited dMSNs are distributed across both compartments [34]. This finding raises the possibility that striosome‐enriched dMSN populations contribute to behavioural updating when alcohol is no longer available. However, whether increasing activity in such a population during extinction is sufficient to facilitate extinction‐related behavioural change and reduce subsequent alcohol seeking remains unknown.

Here, we examined complementary striatal processes associated with extinction‐dependent suppression of alcohol seeking. First, we determined whether extinction reduced subsequent reactivation of acquisition‐recruited DMS dMSNs. We then tested whether maintaining activity in acquisition‐recruited dMSNs during extinction altered extinction‐related responding and later relapse. Finally, motivated by the striosomal enrichment of extinction‐recruited dMSNs, we tested whether increasing activity in MOR‐expressing dMSNs—a striosome‐enriched population—was sufficient to facilitate extinction‐related behavioural change and reduce subsequent alcohol seeking.

## 2 Results

### 2.1 Extinction Training Suppressed Cue‐Induced Relapse to Alcohol Seeking

**FIGURE 1:**
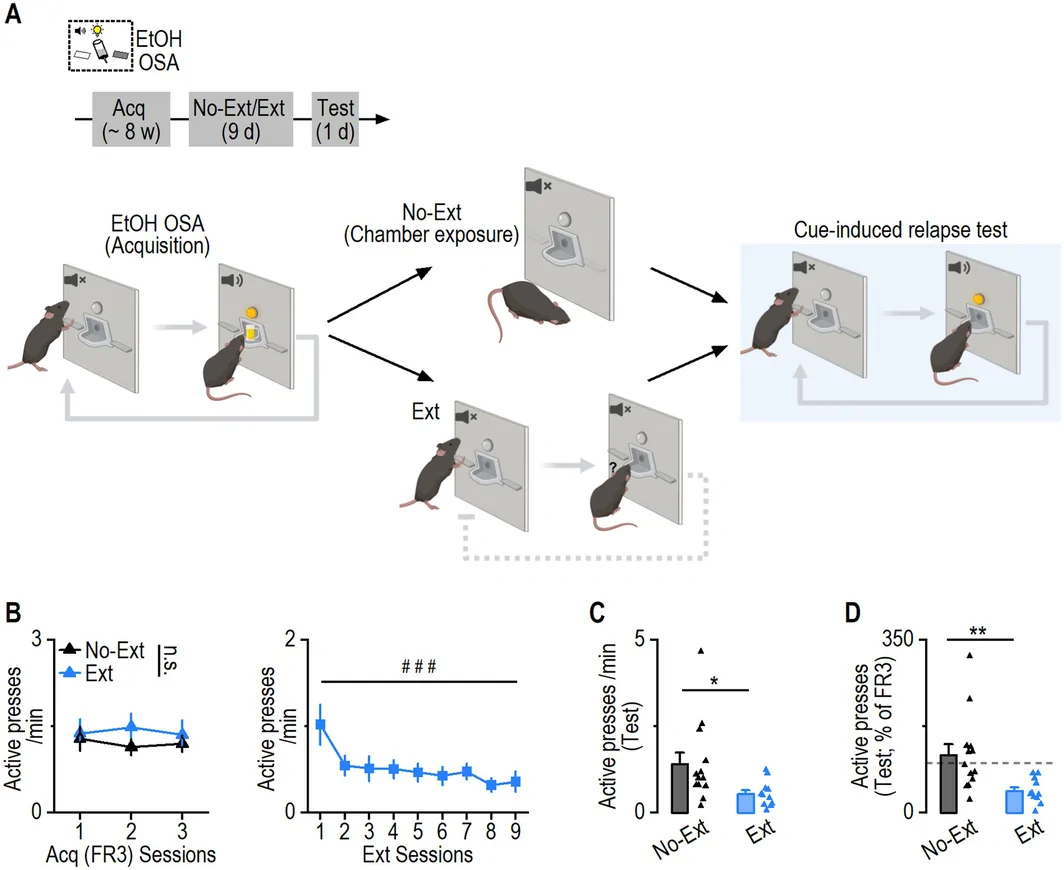
Extinction training suppressed cue‐induced relapse to alcohol seeking. (A) Schematic of the behavioural paradigm. Mice underwent acquisition of operant alcohol self‐administration (Acq; ~8 weeks), followed by either extinction training (Ext; 9 days) or no‐extinction control (No‐Ext). All groups were subsequently tested for cue‐induced relapse. (B) Left: Active lever pressing during the final acquisition sessions (FR3 schedule) was comparable between groups prior to extinction or no‐extinction manipulation. Two‐way RM ANOVA: *F*_(1, 22)_ = 0.579, *p* = 0.455. Right: Active lever responding decreased during extinction and remained at relatively low levels across the nine daily sessions. Because the residuals from the repeated‐measures analysis did not satisfy the normality assumption (Shapiro–Wilk W = 0.963, *p* = 0.0068), extinction responding was analysed using the Friedman's test: between sessions: X^2^ = 28.13, ^###^ *p* = 0.0005. (C) Mice with a history of extinction exhibited reduced raw active lever pressing during relapse test compared to no‐extinction controls. Because the residuals from the two‐group comparison did not satisfy the normality assumption (Shapiro–Wilk W = 0.803, *p* = 0.0003), data were analysed using the Mann–Whitney test: *U* = 29.5, *p = 0.013. (D) Relapse responding expressed as a percentage of acquisition responding demonstrates that extinction training significantly suppressed alcohol relapse. Because the residuals from the two‐group comparison did not satisfy the normality assumption (Shapiro–Wilk W = 0.849, *p* = 0.0022), data were analysed using the Mann–Whitney test: *U* = 22, **p = 0.0031. *n* = 13 mice (No‐Ext; 8 male and 5 female) and 11 mice (Ext; 6 male and 5 female).

Previous studies showed that extinction training reduces cue‐ or context‐induced reinstatement of other drug‐seeking behaviours [8, 53, 54, 55]. We thus first asked whether operant alcohol extinction suppresses cue‐induced relapse of alcohol seeking. Wild‐type mice were trained in an operant alcohol self‐administration paradigm for ~8 weeks (Figure 1A). The chamber contained retractable active and inactive levers flanking a central magazine, with a cue light positioned above the magazine. Under a fixed ratio 1 (FR1) schedule, each active lever press resulted in alcohol delivery paired with a cue light and tone, followed by a 20‐s timeout. Lever presses on either the active or inactive lever during the timeout were recorded but had no programmed consequences. The training schedule progressively advanced to FR2 and then FR3. Mice achieved stable responding during the final FR3 sessions (Figure 1B, left; two‐way RM ANOVA: *F*_(1, 22)_ = 0.579, *p* = 0.455; Figure S1A).

Following acquisition, mice were divided into two groups. One group underwent 9 days of lever extinction, during which active lever presses no longer produced alcohol or cue presentations. Responding decreased markedly during the first extinction session and remained at relatively low levels across the subsequent sessions (Figure 1B, right; Friedman's test: between sessions: X^2^ = 28.13, *p* = 0.0005; Figure S1B). The control group was returned daily to the chamber for the same duration but with levers retracted, controlling for context exposure without actual lever‐extinction learning.

One day after the final extinction session, both groups underwent a cue‐induced relapse test. Raw active lever presses were significantly lower in the extinction group compared to controls (Figure 1C: Mann–Whitney test: *U* = 29.5, *p* = 0.013; Figure S1C). Relapse responding reached 44.25% ± 7.54% of acquisition levels in the extinction group, compared with 117.23% ± 21.94% in controls (Figure 1D: Mann–Whitney test: *U* = 22, *p* = 0.0031). These findings demonstrate that a history of operant extinction training suppresses cue‐induced relapse of alcohol seeking.

### 2.2 Extinction Reduced Reactivation of Acquisition‐Recruited dMSN Ensembles During Relapse

Having established that extinction suppresses relapse behaviour, we next sought to identify the underlying cellular mechanism. Prior studies established that sparse neuronal ensembles in several brain regions causally regulate drug seeking [30, 31, 32, 33]. Building on this literature, our recent work showed that DMS dMSNs recruited during acquisition of alcohol self‐administration form ensembles that encode alcohol‐related memories, and that their reactivation drives relapse [34]. We therefore hypothesized that extinction training suppresses relapse by reducing reactivation of these acquisition‐recruited dMSN ensembles.

**FIGURE 2:**
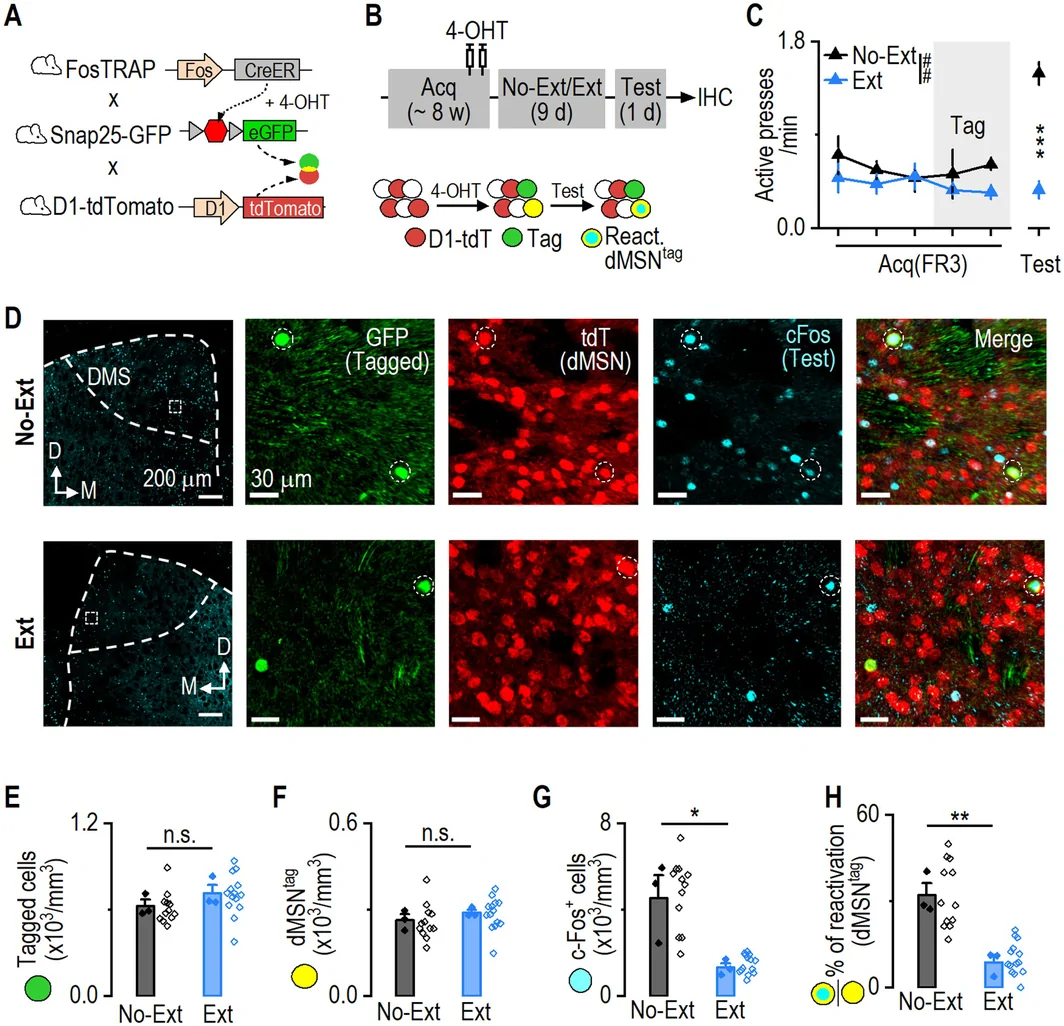
Extinction reduced reactivation of acquisition‐recruited dMSN ensembles during relapse. (A) Mechanism of activity‐dependent GFP expression in tagged neurons and tdTomato (tdT) expression in dMSNs of FosTRAP;Snap25‐GFP;D1‐tdT mice. (B) Behavioural and tagging timeline illustrating acquisition, extinction or no‐extinction, relapse testing and tissue collection for ensemble reactivation analysis using c‐Fos immunohistochemistry (IHC). dMSNs express tdT. After tagging, tagged dMSNs (dMSN^tag^) co‐express Snap25‐GFP and D1‐tdT. During relapse, reactivated dMSN^tag^ were labelled with c‐Fos IHC. (C) Raw active lever pressing during final five FR3 sessions and relapse test in extinction and no‐extinction groups. Two‐way RM ANOVA: *F*_(5, 20)_ = 4.981, ^##^ *p* = 0.0040; Sidak's multiple comparisons test for No‐Ext vs. Ext: Acq sessions 1–5: *t* = 1.24, 0.74, 0.083, 0.85, 1.51; *p* = 0.78, 0.98, 0.99, 0.95, 0.61; Test: *t* = 6.318, ***p < 0.001. Mean active lever responding across the final three FR3 sessions did not differ between the No‐Ext and Ext groups. Unpaired *t*‐test: *t*_(4)_ = 1.03, *p* = 0.36. (D) Representative confocal images of dorsomedial striatum showing tagged acquisition neurons (GFP), D1‐tdT labelling and c‐Fos expression following relapse. Insets show higher magnification views. (E) Both groups had similar densities of tagged neurons in the dorsomedial striatum. Mann Whitney test: *U* = 2, *p* = 0.40. (F) Both groups had similar densities of dMSN^tag^. Unpaired *t*‐test: *t*_(4)_ = 1.22, *p* = 0.29. (G) Extinction training reduced the density of c‐Fos^+^ cells during relapse. Unpaired *t*‐test: *t*_(4)_ = 2.97, *p = 0.041. (H) Extinction training reduced the percentage of acquisition‐recruited dMSNs reactivated during relapse. Unpaired *t*‐test: *t*_(4)_ = 4.83, **p = 0.0084. *n* = 3 mice per group (C; all females), 12 slices/3 mice (No‐Ext; E‐H) and 14 slices/3 mice (Ext; E‐H). Bars represent mean ± SEM calculated from animal‐level means. Solid symbols indicate the mean value for each mouse after averaging all sections from that animal, whereas open symbols show individual section‐level measurements for visualization only. All statistical analyses were performed using mice as the experimental unit.

To test this, we generated triple transgenic mice (FosTRAP; Snap25‐GFP; D1‐tdTomato) to label neurons activated during acquisition with GFP and identify dMSNs with tdTomato (Figure 2A). Neurons co‐expressing GFP and tdTomato were defined as acquisition‐tagged dMSNs (dMSN^tag^). Mice were trained for ~8 weeks, and tagging was induced during the final two FR3 sessions via 4‐hydroxytamoxifen (4‐OHT; Figure 2B,C) injection. Mice then underwent either 9 days of extinction or no‐extinction control procedures, followed by cue‐induced relapse testing. Ninety minutes after the test, brains were collected for c‐Fos immunohistochemistry to assess reactivation of acquisition‐tagged dMSNs (GFP^+^ and tdTomato^+^ and c‐Fos^+^).

Both groups displayed comparable acquisition performance, and as expected, relapse responding was reduced in the extinction group (Figure 2C: two‐way RM ANOVA: *F*_(5, 20)_ = 4.981, *p* = 0.0040). The density of tagged neurons and tagged dMSNs did not differ between groups (Figure 2D–F; 2E: Mann Whitney test: *U* = 2, *p* = 0.40; 2F: unpaired *t*‐test: *t*_(4)_ = 1.22, *p* = 0.29), confirming equivalent initial labelling during acquisition. However, extinction markedly reduced the density of c‐Fos^+^ neurons during relapse (Figure 2G: unpaired *t*‐test: *t*_(4)_ = 2.97, *p* = 0.041), and significantly decreased the reactivation rate of tagged dMSNs [(c‐Fos^+^ and GFP^+^ and tdTomato^+^)/(GFP^+^ and tdTomato^+^)] (Figure 2H: unpaired *t*‐test: *t*_(4)_ = 4.83, *p* = 0.0084). To determine whether extinction also altered the relative representation of acquisition‐tagged neurons within the acutely active dMSN population, we calculated the percentage of c‐Fos‐positive dMSNs that had previously been tagged during acquisition. This proportion did not differ significantly between the extinction and no‐extinction groups (Figure S2A; unpaired *t*‐test: *t*_(4)_ = 0.225, *p* = 0.833).

Thus, extinction reduced overall neuronal recruitment and decreased re‐engagement of acquisition‐tagged dMSNs during cue‐induced relapse testing. The unchanged relative representation of acquisition‐tagged neurons among acutely active dMSNs suggests that this effect occurred as part of a broader reduction in dMSN recruitment rather than a selective redistribution of the active population.

### 2.3 Chemogenetic Activation of Acquisition‐Recruited dMSN Ensembles During Extinction Slowed the Reduction in Responding and Promoted Relapse

**FIGURE 3:**
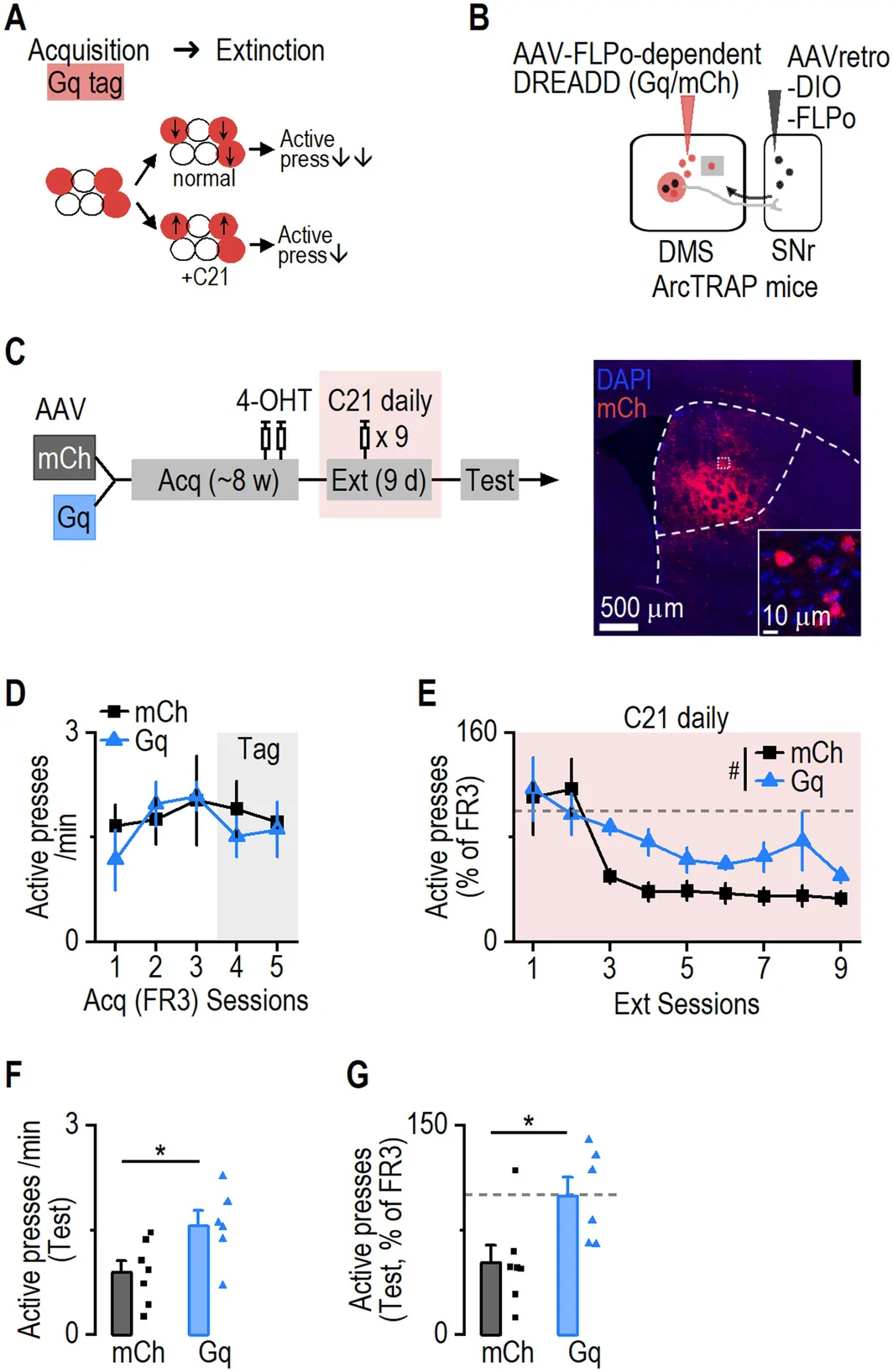
Chemogenetic activation of acquisition‐recruited dMSN ensembles during extinction slowed the reduction in responding and promoted relapse. (A) During extinction, acquisition‐recruited striatal ensembles are suppressed, contributing to the reduction of alcohol‐seeking behaviour. We hypothesized that maintaining activity in acquisition‐recruited dMSNs during extinction would alter extinction‐related responding and increase subsequent relapse. (B) Schematic illustrating intersectional viral approach to express DREADD in acquisition‐recruited striatal dMSNs in ArcTRAP mice. (C) Following viral infusion, mice underwent ~8 weeks of operant alcohol self‐administration, with tagging occurring during the last 2 days of training. Three weeks later, they underwent daily chemogenetic activation (via C21 injection, 1 mg/kg) during extinction sessions, followed by cue‐induced relapse testing without C21 injection. Sample images (right) showing DREADD expression in the DMS. (D) Active lever pressing during acquisition was comparable between groups. Two‐way RM ANOVA: *F*_(1, 11)_ = 0.29, *p* = 0.60. (E) Chemogenetic activation of acquisition‐recruited dMSNs resulted in persistently elevated responding across extinction sessions. Two‐way RM ANOVA: *F*_(1, 11)_ = 5.697, ^#^ *p* = 0.036. (F) Activation of acquisition‐recruited dMSNs during extinction enhanced raw active presses during cue‐induced relapse. Unpaired *t*‐test: *t*_(11)_ = 2.46, *p = 0.032. (G) Relapse responding expressed as a percentage of acquisition responding demonstrates that activation of acquisition ensembles during extinction promotes relapse. Unpaired *t*‐test: *t*_(11)_ = 2.59, *p = 0.025. *n* = 7 mice (mCh; 7 female) and 6 mice (Gq; 5 female and 1 male).

If extinction suppresses subsequent alcohol seeking in part by reducing acquisition‐ensemble activity, then artificially maintaining activity in acquisition‐recruited dMSNs during extinction should interfere with extinction‐related behavioural change and may increase subsequent cue‐induced relapse (Figure 3A).

To selectively manipulate acquisition‐recruited dMSNs, we used ArcTRAP mice combined with a projection‐ and recombinase‐based viral strategy (Figure 3B) [34]. AAVretro‐DIO‐FLPo was injected into the substantia nigra pars reticulata (SNr), enabling retrograde labelling of dMSNs with DIO‐FLPo. FLPo‐dependent designer receptors exclusively activated by designer drugs (DREADDs) [56] were infused into the DMS. In combination with ArcTRAP‐mediated CreER activation during acquisition via 4‐OHT administration, which allowed acquisition‐recruited dMSNs expressing FLPo, this strategy restricted hM3Dq (Gq) or mCherry (mCh) expression to acquisition‐recruited dMSNs.

After stable FR3 performance and tagging during the final acquisition sessions, mice were allowed 3 weeks for viral expression before extinction training (Figure 3C,D; 3D: two‐way RM ANOVA: *F*_(1, 11)_ = 0.29, *p* = 0.60; Figure S1D). During the 9‐day extinction phase, mice received daily Compound 21 (C21; DREADD agonist) injections (1 mg/kg, i.p.) prior to sessions to activate Gq‐expressing neurons. This dose was previously shown to activate hM3Dq‐expressing striatal ensembles using the same ArcTRAP mouse line and intersectional viral strategy employed here [34], while producing minimal effects on locomotor activity and motivated reward‐seeking behaviour in non‐DREADD‐expressing animals [57, 58]. Chemogenetic activation of acquisition‐recruited dMSNs resulted in higher active‐lever responding during the middle‐to‐late extinction sessions compared with mCh controls (Figure 3E: two‐way RM ANOVA: *F*_(1, 11)_ = 5.697, *p* = 0.036). During a subsequent relapse test conducted without C21, Gq mice showed markedly elevated raw active pressing (Figure 3F: unpaired *t*‐test: *t*_(11)_ = 2.46, *p* = 0.032). Normalized relapse responding reached 99.49% ± 13.49% of acquisition levels in Gq mice and 51.91% ± 12.45% in controls (Figure 3G: unpaired *t*‐test: *t*_(11)_ = 2.59, *p* = 0.025). Inactive‐lever responding did not differ between groups during extinction or the subsequent cue‐induced test (Figure S1E,F), indicating that the increase in active‐lever responding was not accompanied by a generalized elevation of lever pressing.

Thus, maintaining activity in acquisition‐recruited dMSNs during extinction altered extinction‐related responding and was followed by greater relapse in the absence of C21 at test.

### 2.4 Chemogenetic Activation of MOR‐Expressing, Striosome‐Enriched dMSNs During Extinction Facilitated Extinction‐Related Behavioural Change and Suppressed Relapse

**FIGURE 4:**
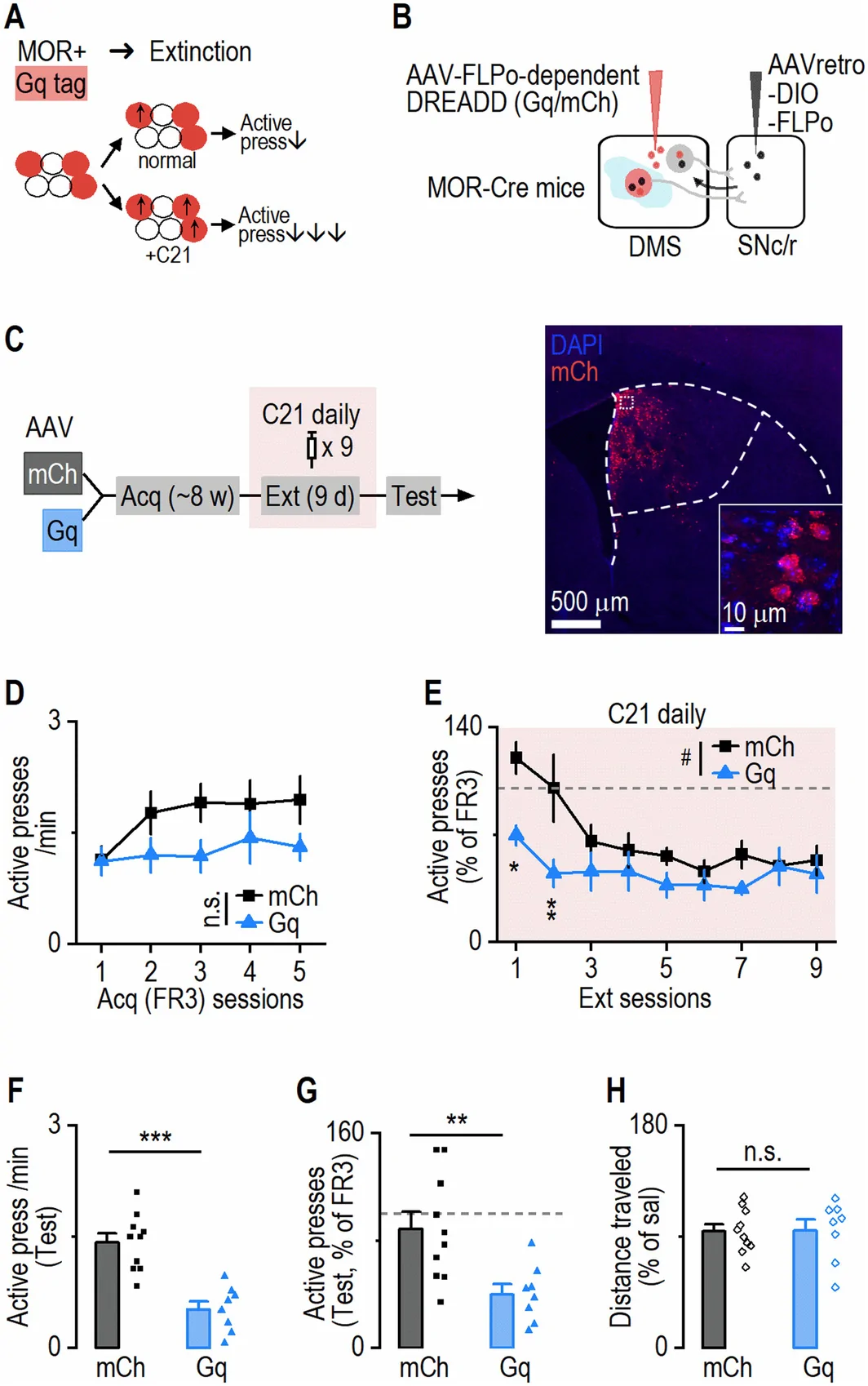
Chemogenetic activation of MOR‐expressing, striosome‐enriched dMSNs during extinction training facilitated extinction‐related behavioural change and suppressed relapse. (A) We hypothesized that increasing activity in MOR‐expressing, striosome‐enriched dMSNs during extinction would facilitate the reduction of alcohol‐seeking behaviour and suppress subsequent cue‐induced responding. (B) Schematic illustrating intersectional viral approach to express DREADD in MOR‐expressing dMSNs in MOR‐Cre mice. (C) Following acquisition and viral expression, mice underwent daily chemogenetic activation during extinction sessions, followed by relapse testing. Sample image (right) showing DREADD expression in the DMS. (D) Active lever pressing during acquisition was comparable between groups. Two‐way RM ANOVA: *F*_(1, 16)_ = 2.28, *p* = 0.15. Mean responding during the final three FR3 sessions did not differ significantly between groups (unpaired *t*‐test: *t*_(16)_ = 1.382, *p* = 0.186). (E) Activation of MOR‐expressing dMSNs accelerated extinction learning and reduced responding across extinction sessions. Two‐way RM ANOVA: Group × Session: *F*_(8, 128)_ = 2.41, ^#^ *p* = 0.0185. Sidak's multiple comparisons test for mCh vs. Gq: session 1: *t* = 3.23, *p = 0.014; session 2: *t* = 3.53, **p = 0.0049. A complementary ANCOVA of raw mean extinction responding, with mean responding during the final three FR3 sessions included as a covariate, confirmed a significant baseline‐adjusted group effect (*F*_(1, 15)_ = 12.51, *p* = 0.003). (F) Activation of MOR‐expressing dMSNs during extinction reduced raw active presses during relapse. Unpaired *t*‐test: *t*_(16)_ = 5.39, ***p < 0.001. (G) Relapse responding expressed as a percentage of acquisition responding demonstrates that activation of MOR‐expressing dMSNs during extinction suppressed relapse. Unpaired *t*‐test: *t*_(16)_ = 3.5, **p = 0.0029. (H) Chemogenetic activation of MOR‐expressing dMSNs did not alter distance travelled in an open field test. Unpaired *t*‐test: *t*_(16)_ = 0.065, *p* = 0.95. *n* = 10 mice (mCh; 2 female and 8 male) and 8 mice (Gq; 8 female).

In addition to reducing the influence of acquisition‐recruited ensembles, extinction requires behavioural updating when lever pressing no longer produces alcohol. Our recent work showed that acquisition‐recruited dMSNs are distributed across both matrix and striosome compartments, whereas dMSNs recruited during extinction are enriched in striosomes [34]. Thus, these populations are not defined by a strict matrix‐versus‐striosome division, but extinction‐recruited dMSNs exhibit a relative striosomal bias. This finding raised the possibility that striosome‐enriched output neurons contribute to extinction‐related behavioural updating. To test whether increasing activity in this broader population is sufficient to facilitate extinction‐related behavioural change, we used an intersectional chemogenetic strategy to target MOR‐expressing DMS dMSNs, a striosome‐enriched population. We hypothesized that activation of this population during extinction would facilitate the reduction of alcohol‐seeking behaviour and suppress subsequent cue‐induced responding (Figure 4A).

To target a MOR‐defined, striosome‐enriched dMSN population, we used MOR‐Cre mice in combination with the same intersectional dual‐virus strategy (Figure 4B). AAVretro‐DIO‐FLPo was infused into the substantia nigra pars compacta (SNc)/SNr, enabling retrograde labelling of striatal dMSNs. Because FLPo expression was Cre‐dependent, FLPo expression was restricted to MOR‐Cre‐expressing striatal neurons projecting to the SNc/SNr, whereas other dMSNs were excluded. Subsequent infusion of an FLPo‐dependent hM3Dq (Gq) or mCherry virus into the DMS restricted DREADD expression specifically to MOR‐expressing, striosome‐enriched dMSNs. After mice achieved stable performance under the FR3 schedule (Figure 4C,D: two‐way RM ANOVA: *F*_(1, 16)_ = 2.28, *p* = 0.15; Figure S1G), they underwent 9 days of extinction training with daily C21 administration to activate Gq‐expressing dMSNs.

Chemogenetic activation of MOR‐expressing, striosome‐enriched dMSNs significantly accelerated the reduction in active‐lever responding, particularly during the early extinction sessions, as compared to mCh controls (Figure 4E: two‐way RM ANOVA: Group × Session: *F*_(8, 128)_ = 2.41, *p* = 0.0185). During the subsequent cue‐induced relapse test (without C21), Gq‐expressing mice showed significantly lower raw active responding and reduced normalized responding compared to controls (Figure 4F,G; 4F: unpaired *t*‐test: *t*_(16)_ = 5.39, *p* < 0.001; 4G: unpaired *t*‐test: *t*_(16)_ = 3.5, *p* = 0.0029). Inactive‐lever responding remained low and did not differ between groups during extinction or the subsequent cue‐induced test (Figure S1H,I), indicating that the reduction in active‐lever responding was not accompanied by a generalized suppression of lever pressing. Importantly, activation of these dMSNs did not alter locomotor activity in an open‐field test (Figure 4H: unpaired *t*‐test: *t*_(16)_ = 0.065, *p* = 0.95), indicating that the behavioural effects were not attributable to non‐specific motor changes.

Together, these findings show that chemogenetically increasing activity in MOR‐expressing, SNc/SNr‐projecting dMSNs during extinction accelerated the reduction in active‐lever responding and was followed by lower cue‐induced responding in the absence of C21. Thus, mice receiving chemogenetic activation of this MOR‐defined, striosome‐enriched population showed facilitated extinction‐related behavioural change and reduced subsequent alcohol seeking.

## 3 Discussion

In this study, we identify complementary striatal processes associated with extinction‐dependent suppression of cue‐induced alcohol seeking. Extinction reduced subsequent reactivation of acquisition‐recruited dorsomedial striatal dMSN ensembles. Conversely, maintaining activity in acquisition‐recruited dMSNs during extinction slowed the reduction in alcohol‐seeking responses and enhanced later relapse. Increasing activity in MOR‐expressing, SNc/SNr‐projecting dMSNs during extinction facilitated the reduction in responding and decreased subsequent relapse. Together, these findings suggest that extinction reduces the influence of previously established alcohol‐memory ensembles, and that increasing activity in an additional MOR‐expressing dMSN population can support behavioural updating. This population‐level reorganization is consistent with prior evidence that reward‐promoting and reward‐omission or extinction ensembles can coexist within the same region and exert opposing control over behaviour [15, 16, 39, 40].

### 3.1 Extinction Suppresses Reactivation of Acquisition‐Recruited dMSN Ensembles

A central finding of this study is that extinction training reduced reactivation of acquisition‐recruited dMSN ensembles during cue‐induced relapse. More broadly, alcohol‐associated memories recruit cue‐responsive neuronal populations and activity‐dependent molecular programmes across infralimbic and medial prefrontal, hippocampal and amygdalar circuits, and manipulation of these populations or memory‐reconsolidation processes can alter subsequent alcohol seeking [32, 59, 60, 61]. Within the DMS, our previous work demonstrated that dMSNs recruited during operant alcohol self‐administration encode alcohol‐associated memories, and that their reactivation promotes relapse‐like behaviour [34]. Here, extinction decreased the probability that acquisition‐tagged dMSNs were reactivated during relapse, in parallel with reduced alcohol seeking. However, the proportion of acutely active dMSNs that had previously been tagged during acquisition was unchanged. Thus, acquisition‐tagged neurons retained a similar relative representation within the remaining active dMSN population. These findings indicate that extinction dampens re‐engagement of the acquisition ensemble as part of a broader reduction in striatal recruitment, rather than producing a detectable selective exclusion of acquisition‐tagged neurons from the active population.

Maintaining activity in acquisition‐recruited dMSNs during extinction produced higher responding during the middle‐to‐late extinction sessions and greater relapse in a subsequent test conducted without C21. The later behavioural difference therefore cannot be attributed to an acute effect of chemogenetic activation at the time of testing. Nevertheless, the greater responding experienced during extinction may itself have contributed to the subsequent relapse difference. The present design cannot distinguish whether the manipulation directly disrupted extinction‐memory formation or altered the behavioural experience during extinction in a manner that increased later seeking. Therefore, this experiment demonstrates that maintaining activity in acquisition‐recruited dMSNs during extinction is sufficient to alter extinction‐related behaviour and later relapse, but it does not establish that endogenous suppression of this population is necessary for extinction.

The observational and causal experiments used FosTRAP and ArcTRAP mice, respectively, to access acquisition‐recruited dMSNs. Because Arc and c‐Fos expression can identify partially overlapping but non‐identical neuronal populations [62], the two approaches cannot be assumed to label the same individual neurons. Nevertheless, both systems tagged DMS dMSNs activated during the same operant alcohol‐acquisition phase and therefore sample the broader acquisition‐recruited dMSN ensemble. Experiments 2 and 3 thus provide complementary observational and causal evidence at the population level, although direct cell‐by‐cell correspondence between the FosTRAP‐ and ArcTRAP‐defined populations was not determined.

The present findings are consistent with a broader addiction‐ensemble framework in which sparse, behaviourally recruited neuronal populations exert distinct or opposing control over drug seeking. Causal drug‐seeking ensembles have been identified in the ventral mPFC during heroin relapse, the nucleus accumbens shell during cocaine reinstatement, the DMS during incubated methamphetamine seeking and the infralimbic cortex during alcohol seeking [30, 31, 32, 33]. Complementary studies showed that reward‐omission or extinction ensembles in the infralimbic cortex and vmPFC suppress reward and drug seeking [15, 16, 39, 40]. In alcohol models, inhibition of acquisition‐related DMS ensembles facilitates extinction and suppresses subsequent relapse [35], and our recent study demonstrated that acquisition‐ and extinction‐recruited dMSN ensembles coexist within the DMS and exert opposing control over alcohol seeking [34]. The present findings extend this framework by showing that extinction also reduces subsequent reactivation of acquisition‐recruited dMSNs.

These results are more consistent with reduced accessibility of the original alcohol memory than with memory erasure. Acquisition‐tagged neurons remained anatomically present but were less likely to be re‐engaged during relapse. Drug seeking and drug refraining have been associated with synaptic potentiation in distinct populations of nucleus accumbens medium spiny neurons [53], and broader engram studies indicate that memory identity and retrieval depend on ensemble‐specific synaptic strength and excitability [63, 64, 65]. Extinction can also induce depotentiation or weakening of synapses supporting the original association [66, 67, 68]. Extinction may therefore reduce the ability of alcohol‐associated inputs to recruit acquisition‐tagged dMSNs by modifying synaptic strength, intrinsic excitability or both, thereby limiting behavioural expression of the original memory without eliminating its underlying neuronal representation.

### 3.2 Extinction as Active Striatal Learning: Contribution of MOR‐Expressing, Striosome‐Enriched dMSNs

In addition to reducing the influence of acquisition‐encoded ensembles, extinction is an active learning process in which animals update the expected consequences of a previously reinforced action [2, 69]. Emerging evidence suggests that striosomal neurons participate in behavioural updating, decision conflict and negative reinforcement [42, 44, 70, 71]. Consistent with a functional contribution of this circuitry, chemogenetic activation of MOR‐expressing, SNc/SNr‐projecting dMSNs during extinction accelerated the reduction in active‐lever responding and resulted in lower relapse during subsequent cue exposure. Because C21 was not administered during the later test, the reduction in relapse was not caused by an acute suppressive effect of DREADD activation at the time of testing. Inactive‐lever responding remained low and comparable between groups, and locomotor activity was unchanged, indicating that the behavioural effects were not attributable to generalized motor suppression or non‐specific reductions in lever pressing.

The MOR‐based manipulation was not activity dependent and therefore did not selectively target the endogenous dMSN ensemble recruited during extinction. Instead, it increased activity in a broader population of MOR‐expressing, SNc/SNr‐projecting DMS dMSNs enriched in the striosomal compartment, irrespective of whether individual neurons were naturally active during extinction. The experiment therefore establishes that increasing activity in this population is sufficient to facilitate extinction‐related behavioural change. Although striatal neurons can encode action–outcome relationships [72], the present experiment does not demonstrate preferential endogenous recruitment, the specific information encoded by the manipulated neurons or their necessity for extinction. Moreover, because the population was defined by MOR expression and projection pattern, the present design cannot separate the contributions of MOR‐defined cellular identity from striosomal localization. Acquisition‐recruited dMSNs are also distributed across both matrix and striosome compartments [34] and may therefore partially overlap with the MOR‐defined population manipulated here.

An additional limitation of Experiment 4 is the unequal sex distribution between viral groups. All hM3Dq mice were female, whereas the mCherry group contained eight males and two females. Consequently, sex and viral treatment were strongly confounded, and the present experiment cannot determine whether the observed behavioural differences reflect chemogenetic activation alone, a contribution of sex or an interaction between these factors. Therefore, the Figure 4 findings should not be interpreted as demonstrating a sex‐independent effect of MOR‐expressing dMSN activation. Replication using sex‐balanced treatment groups will be required to establish whether this effect generalizes across sexes.

MOR‐expressing, striosome‐enriched dMSNs are anatomically positioned to influence behavioural updating through the midbrain dopamine system. Whereas canonical dMSNs primarily project to basal ganglia output nuclei such as the SNr, subsets of striosomal projection neurons form specialized striosome–dendron ‘bouquet’ structures that contact SNc dopamine neurons and their ventrally extending dendrites [43, 73, 74, 75, 76]. Alcohol may modify this circuitry through the endogenous opioid system. Acute alcohol exposure increases endogenous opioid signalling [77], whereas repeated alcohol exposure can reduce MOR expression or availability in the striatum and other regions [78]. Because MOR activation suppresses GABAergic output from MOR‐expressing striatal dMSNs [52], reduced MOR signalling after repeated alcohol exposure could diminish this presynaptic inhibition and permit stronger output from MOR‐expressing, striosome‐enriched neurons.

During extinction, omission of the expected alcohol outcome generates a negative reward‐prediction error. Reward omission recruits neuronal ensembles in infralimbic and ventromedial prefrontal circuits [15, 16], whereas striosomes receive preferential input from limbic/associational and medial prefrontal cortical regions [73, 74]. These observations raise the possibility that omission‐responsive cortical activity recruits MOR‐expressing, striosome‐enriched dMSNs during extinction. Consequently, increased inhibitory output from these dMSNs could reduce SNc dopamine activity and nigrostriatal dopamine signalling, thereby lowering the expected value of lever pressing and weakening selection of the previously reinforced action. This model is consistent with our previous observation that extinction training reduced action‐specific striatal dopamine transients during subsequent relapse [5]. However, this proposed mechanism remains speculative because the present study did not measure dopamine dynamics during extinction or isolate the causal contribution of the striosome–SNc projection.

### 3.3 Coordinated Striatal Mechanisms Underlying Extinction‐Dependent Suppression of Relapse

Taken together, our findings suggest that extinction modifies the influence of differentially defined, potentially overlapping DMS dMSN populations. The distinction between these populations is not a strict matrix‐versus‐striosome division: Acquisition‐recruited dMSNs occur in both compartments, whereas extinction‐recruited dMSNs show a relative striosomal enrichment [34]. Extinction reduced the probability that acquisition‐recruited dMSNs were reactivated during relapse, whereas chemogenetic activation of MOR‐expressing, SNc/SNr‐projecting dMSNs was associated with reduced alcohol‐seeking behaviour during extinction and subsequent relapse. These processes may interact within striatal networks to reduce the influence of previously reinforced actions while supporting behavioural updating when alcohol is no longer available.

These findings extend ensemble‐based models of extinction beyond cortical, hippocampal and amygdalar circuits by identifying the dorsal striatum as an additional site in which defined projection‐neuron populations regulate alcohol‐memory expression and behavioural updating. Rather than demonstrating that the two manipulated populations are anatomically exclusive or that either population alone mediates extinction, the results support a framework in which extinction alters the balance among functionally defined striatal populations. This framework provides a basis for understanding how striatal ensemble dynamics contribute to relapse suppression and may inform strategies designed to strengthen extinction‐based interventions.

## 4 Materials and Methods

### 4.1 Animals

Drd1a‐tdTomato (#016204), Snap25‐LSL‐F2A‐GFP (#021879), FosTRAP (Fos^2A‐iCreER^; #030323) and ArcTRAP (Arc^CreER^; #021881) [52, 79, 80] mice were purchased from the Jackson Laboratory. MOR‐Cre mice were provided by Dr. Brigitte Kieffer. FosTRAP mice were crossed with Snap25‐GFP and D1‐tdTomato mice to obtain FosTRAP;Snap25‐GFP;D1‐tdTomato mice. Genotypes were determined by PCR analysis of Cre or the fluorescent protein gene in tail DNA. Male and female mice were included in the study. Animals were assigned to experimental groups without selection based on sex. Because several experiments required specific combinations of transgenic alleles, the sex distribution of available offspring was sometimes uneven, and sex‐balanced group assignment was not always feasible. Within each experiment, animals were approximately age‐matched and randomly assigned to experimental groups when possible. All mice were 3–8 months old. The numbers of male and female mice are reported in the corresponding figure legends. Viral group assignment was determined before behavioural training, with animals matched as closely as possible for age. Because viral infusion preceded operant acquisition, animals could not be retrospectively assigned or matched according to acquisition performance. Animals were housed in a temperature‐ and humidity‐controlled vivarium with a reversed 12‐h light/dark cycle (lights on at 10:00 PM and off at 10:00 AM). All behaviour experiments were conducted during the dark phase, starting approximately 1 h after the light went off. Food and water were available ad libitum. All animal care and experimental procedures were approved by the Texas A&M University Institutional Animal Care and Use Committee.

### 4.2 Experimental Design and Group Composition

#### 4.2.1 Experiment 1: Effect of Extinction on Cue‐Induced Alcohol Seeking

Wild‐type mice were trained in operant alcohol self‐administration and assigned after acquisition to either a no‐extinction control group or an extinction group. The no‐extinction group comprised 13 mice (eight male and five female), and the extinction group comprised 11 mice (six male and five female). Extinction mice underwent nine daily lever‐extinction sessions, whereas no‐extinction controls received equivalent daily exposure to the operant chamber with the levers retracted. Both groups underwent cue‐induced testing 1 day after the final extinction or context‐exposure session.

#### 4.2.2 Experiment 2: Reactivation of Acquisition‐Recruited dMSNs After Extinction

FosTRAP;Snap25‐GFP;D1‐tdTomato triple‐transgenic mice were used to label acquisition‐recruited neurons with GFP and identify dMSNs by D1‐tdTomato expression. Mice received 4‐OHT immediately after each of the final two FR3 acquisition sessions. Animals were then assigned to no‐extinction or extinction groups, with three female mice in each group. Extinction mice underwent nine daily lever‐extinction sessions, whereas no‐extinction mice did not. Both groups underwent cue‐induced testing 1 day later, and tissue was collected 90 min after the test for c‐Fos immunohistochemistry.

#### 4.2.3 Experiment 3: Chemogenetic Activation of Acquisition‐Recruited dMSNs During Extinction

ArcTRAP mice received bilateral infusion of AAVretro‐DIO‐FLPo into the SNr and either AAV‐fDIO‐hM3Dq‐mCherry or AAV‐fDIO‐mCherry into the DMS. Acquisition‐recruited neurons were tagged by administering 4‐OHT immediately after the final two FR3 acquisition sessions. Animals were then allowed 3 weeks for recombination and DREADD expression before extinction training. The final mCherry control group comprised seven female mice, and the hM3Dq group comprised six mice (five female and one male). Both groups received C21 at 1 mg/kg, intraperitoneally, 30 min before each of the nine extinction sessions. Cue‐induced testing was conducted 1 day after the final extinction session without C21 administration.

#### 4.2.4 Experiment 4: Chemogenetic Activation of MOR‐Expressing, Striosome‐Enriched dMSNs During Extinction

MOR‐Cre mice received bilateral infusion of AAVretro‐DIO‐FLPo into the SNc/SNr and either AAV‐fDIO‐hM3Dq‐mCherry or AAV‐fDIO‐mCherry into the DMS. This intersectional strategy restricted expression to MOR‐expressing DMS neurons projecting to the SNc/SNr. Viral group assignment occurred before operant behavioural training. The final mCherry control group comprised 10 mice (eight male and two female), and the hM3Dq group comprised eight female mice. Because of this unequal sex distribution, sex and viral treatment could not be independently evaluated in Experiment 4. Both groups received C21 at 1 mg/kg, intraperitoneally, 30 min before each of the nine extinction sessions. Cue‐induced testing was conducted without C21 1 day after the final extinction session. Locomotor activity was subsequently evaluated following saline and C21 administration using the open‐field procedure described below.

Across Experiments 3 and 4, mice that failed to acquire operant alcohol self‐administration according to the prespecified acquisition criterion were excluded before extinction training. Two hM3Dq mice from Experiment 3 and one hM3Dq mouse from Experiment 4 were excluded on this basis. No animals were excluded according to extinction or cue‐induced test performance, and no additional animals or data points were removed.

### 4.3 Method Details

#### 4.3.1 Intermittent Access to 20% Alcohol Two‐Bottle‐Choice Drinking Procedure

To provide prior exposure to 20% alcohol before operant self‐administration training, we used an intermittent‐access two‐bottle‐choice (2BC) drinking procedure [20, 26, 29, 81, 82, 83, 84, 85, 86, 87]. Mice were given free access to two bottles containing water or 20% alcohol for three 24‐h sessions (Mondays, Wednesdays and Fridays), with 24‐ or 48‐h withdrawal periods (Tuesdays, Thursdays, Saturdays and Sundays) each week. During the withdrawal periods, the mice had unlimited access to water bottles. The placement of the alcohol bottle was alternated for each drinking session to control side preferences. For all animals that underwent operant self‐administration of alcohol, 3–6 weeks of 2bc were used to acclimate them to alcohol; individual alcohol intake during this pre‐exposure phase was not quantified.

#### 4.3.2 Operant Self‐Administration of Alcohol

Animals were trained to self‐administer 20% alcohol in operant chambers using a fixed ratio (FR) schedule [88, 89, 90]. The mouse operant set‐up (Med Associates) included two levers in each chamber: active and inactive, on the opposite sides of the same wall.

During alcohol operant self‐administratin (OSA) training sessions, a house light centred above the operant chamber was illuminated. Each operant experiment commenced with continuous reinforcement (FR1) every other day for approximately 2–3 weeks, wherein pressing the active lever resulted in an ~0.0186‐mL delivery of 20% alcohol. Actions on the inactive side were documented but did not initiate a programmed event. The alcohol solution was dispensed into a stainless‐steel reservoir situated within the magazine port between active and inactive levers. Each alcohol delivery persisted for 3 s and was accompanied by a tone and a discrete yellow cue light in the magazine port for the same duration. There was a 20‐s timeout period for each delivery, during which active/inactive presses were recorded but did not cause any further alcohol deliveries. When animals were able to achieve at least 10 deliveries under the FR1 schedule, the criterion to receive the alcohol escalated to FR2 (around 1–2 weeks), and eventually progressed to FR3 (around 2 weeks). Each operant session for mice lasted 30 min or 1 h, depending on the learning condition. Because alcohol was delivered into a fixed magazine receptacle and residual solution and spillage could not be quantified reliably, actual alcohol consumption in g/kg could not be determined. Mice that failed to acquire operant alcohol self‐administration were excluded before extinction training and subsequent behavioural analyses. Failure to acquire was defined as failure to earn at least 10 alcohol deliveries under the FR1 schedule within the designated acquisition period. Based on this criterion, two hM3Dq mice from the acquisition‐ensemble experiment shown in Figure 3 and one hM3Dq mouse from the MOR‐Cre experiment shown in Figure 4 were excluded. No other animals or data points were excluded from the study.

##### 4.3.2.1 Extinction Training

The extinction training was conducted under the FR3 schedule. However, pressing on the active side did not result in actual alcohol delivery or cue presentation. Each extinction session for mice lasted 30 min, and in total lasted for 9 days. Nine daily extinction sessions were used based on established operant alcohol‐seeking protocols and to ensure stable low responding before cue‐induced testing [34, 89, 91]. For chemogenetic manipulations during the extinction, C21 (i.p., 1 mg/kg) was injected 30 min prior to the start of the extinction session.

##### 4.3.2.2 Cue‐Induced Relapse Test

In the conventional reinstatement model, reinstatement refers specifically to the return of drug seeking after extinction. Because Experiment 1 included both extinction‐trained and no‐extinction groups, we use the broader term ‘cue‐induced relapse test’ for the common behavioural endpoint. Here, relapse refers operationally to renewed alcohol‐seeking responses elicited by alcohol‐associated cues in the absence of alcohol delivery. The cue‐induced relapse test (30 min) was conducted as previously described [59]. It was carried out under the FR3 schedule, where three actions on the active side triggered the illumination of the cue light within the magazine port, the tone and the pump action sound, with the exception that the first cue presentation was given immediately after the first active press.

#### 4.3.3 Tag Active Neurons

4‐OHT was prepared, and tagging was conducted as previously described [52, 80, 92]. The 4‐OHT solution was freshly prepared on the tagging day. In brief, 4‐OHT powder was dissolved in 200 proof ethanol (20 mg/mL) at room temperature, with continuous shaking until the 4‐OHT was fully dissolved. An equal volume of a 1:4 mixture of castor oil and sunflower seed oil was added, resulting in a final concentration of 10‐mg/mL 4‐OHT. The ethanol was removed by centrifugation under vacuum. The final 4‐OHT in oil was stored at 4°C for a maximum of 12 h before use.

For tagging during acquisition of operant learning, mice were habituated to i.p. injection of saline immediately after each operant session for two consecutive days. On the third and fourth operant sessions (Tag), mice were introduced to an isoflurane chamber immediately after the operant session, and 4‐OHT was i.p. injected at 50 mg/kg. The use of isoflurane was to reduce the pain and discomfort due to 4‐OHT/oil mix injection. After the injection, mice were returned to their home‐cage and left without any disturbance for the following 6 h.

#### 4.3.4 Stereotaxic Virus Infusion

Where required for the experimental design, AAVretro‐DIO‐FLPo (0.6 μL/site) was bilaterally infused into the SNr (AP: −3.15 mm; ML: ±1.35 mm; DV: −4.6 mm), and AAV‐fDIO‐mCherry/AAV‐fDIO‐hM3Dq‐mCherry (0.5 μL/site) was bilaterally infused into the DMS (AP1: +0.98 mm; ML1: ±1.25 mm; DV1: −2.9 to −3.1 mm; AP2: +0.3 mm; ML2: ±1.87 mm; DV2: −2.9 to −3.1 mm) of ArcTRAP or MOR‐Cre mice. Animals were anaesthetised with 3%–4% isoflurane at 1.0 L/min and mounted in a stereotaxic surgery frame. The head was levelled, and craniotomy was performed using stereotaxic coordinates adapted from the brain atlas [93]. The virus was infused at a rate of 0.08 μL/min. At the end of the infusion, the injectors remained at the injection site for an additional 10–15 min before removal to allow for virus diffusion. The scalp incision was then sutured, and the animals were returned to their home cage for recovery.

#### 4.3.5 Open Field Locomotor Activity Test

A transparent open field activity chamber (Med Associates, 43 cm × 43 cm × 21 cm height) was equipped with an infrared beam detector that was connected to a computer. All tests lasted for 10 min. Animals were first habituated in the chamber to remove the novelty‐induced effect. Then, animals were tested for locomotor activity by placing them into the chamber 30 min after i.p. injections of saline. One day later, animals were re‐introduced to the chamber 30 min after i.p. injections of C21 (1 mg/kg). Locomotor activity during the C21‐injected day was compared between groups by normalizing the distance travelled in the C21‐injected day to the distance travelled in the saline‐injected day.

#### 4.3.6 Histology and Cell Counting

Animals were perfused intracardially with 4% formaldehyde in phosphate‐buffered saline (PBS). The brains were removed and post‐fixed overnight in 4% formaldehyde in PBS at 4°C prior to dehydration in 30% sucrose solution. The brains were then cut into 50‐μm coronal sections using a cryostat. c‐Fos IHC was carried out on free‐floating sections. All incubations (except for the primary antibody) and rinses took place at room temperature on a shaker. Sections were rinsed in PBS three times between steps. Sections were first placed in blocking solution with 10% bovine serum albumin (BSA) in PBS with 0.3% triton‐X (PBST) for 1 h and then incubated in primary antibody (rabbit anti‐c‐Fos: for mice: 1:2000, EMD‐Millipore, #PC38) diluted in blocking solution (1% BSA in 0.3% PBST) overnight at 4°C. Sections were then incubated in biotinylated donkey anti‐rabbit (1:500, Jackson Immuno Research) in blocking solution for 1 h. Sections were visualized via incubation for 1 h in Streptavidin‐conjugated Alexa 647 (1:1000, Thermo Fisher) in PBS. After incubation, all slices were washed three times in PBS and mounted.

All images were acquired using a confocal microscope (FluoView 3000, Olympus, Tokyo, Japan) and analysed using IMARIS 8.3.1 (Bitplane, Zürich, Switzerland), as previously reported [25, 94]. Coronal sections were cut at a thickness of 50 μm. For each section analysed from FosTRAP mice, a confocal z‐stack centred within the section was acquired using five optical planes separated by a 3‐μm z‐step, corresponding to a total z‐range of 12 μm. Cells were identified and counted as three‐dimensional objects across the complete z‐stack using the IMARIS, such that cells extending across multiple optical planes were counted once.

Quantification was restricted to an approximately circular ROI positioned consistently at the dorsomedial boundary of the DMS, with a target radius of approximately 300 μm. Because the exact ROI area could vary slightly among images, the actual area reported by IMARIS was recorded for each ROI. The sampled volume was calculated as the IMARIS‐reported ROI area multiplied by the 12‐μm z‐stack depth. Cell density was calculated as the number of three‐dimensionally identified cells divided by the corresponding sampled volume and expressed as cells/mm^3^. These values represent volumetric cell densities within the sampled DMS ROI and not stereological estimates of total DMS cell number.

#### 4.3.7 Quantification and Statistical Analysis

All data are presented as mean ± SEM, with individual animals shown whenever possible. Statistical analyses were conducted using SigmaPlot. Normality was evaluated using the Shapiro–Wilk test on residuals from the corresponding statistical model. For comparisons between two independent groups, residuals were calculated after accounting for the group means and pooled across groups. For repeated‐measures analyses, residuals from the repeated‐measures model were evaluated. Parametric tests were used when the model residuals satisfied the normality assumption. When the residuals showed a significant departure from normality (Shapiro–Wilk test, *p* < 0.05), the corresponding nonparametric test was used.

Two independent groups were compared using a two‐tailed unpaired Student's *t*‐test when the normality assumption was satisfied or a two‐tailed Mann–Whitney *U* test when it was not. Repeated measurements from the same animals were analysed using repeated‐measures ANOVA when its assumptions were satisfied or the Friedman test when the repeated‐measures model residuals did not satisfy the normality assumption. When appropriate, ANOVA or Friedman tests were followed by the multiple‐comparison procedures specified in the corresponding figure legends. For the Figure 4 extinction analysis, an ANCOVA was performed on mean raw extinction responding, with mean active‐lever responding during the final three FR3 sessions included as a covariate. Homogeneity of regression slopes was evaluated using the group × baseline interaction. Exact statistical tests, sample sizes, test statistics, degrees of freedom and *p* values are reported in the figure legends. Statistical significance was defined as *p* < 0.05.

## Author Contributions

Conceptualization: J.W. and X.X. Methodology: X.X. and J.W. Investigation and formal analysis: X.X. Writing – original draft: X.X. Writing – review and editing: J.W. and X.X. Resources: X.W. Funding acquisition: J.W. and X.X. Supervision: J.W.

## Funding

This research was supported by NIAAA (Grants R01AA021505, R01AA027768, U01AA025932, R01AA030293, R01AA032498) and X‐grant from Texas A&M University to J.W., McGovern Fellowship from Texas Research Society on Alcoholism (TRSA) to X.X. and Doctoral Student Small Grant from Research Society on Alcohol (RSA) to X.X.

## Conflicts of Interest

The authors declare no conflicts of interest.

## Supporting information


**Figure S1:** Inactive‐lever responding across acquisition, extinction and cue‐induced relapse tests (A–C). Inactive‐lever responding for the Ext and No‐Ext groups corresponding to the behavioural experiment shown in Figure 1. (A) Inactive‐lever presses during the final three FR3 acquisition sessions. Two‐way RM ANOVA: *F*_(1, 22)_ = 0.73, *p* = 0.399. (B) Inactive‐lever presses across the nine extinction sessions in the Ext group. (C) Inactive‐lever presses during the cue‐induced test in the Ext and No‐Ext groups. Mann–Whitney test: *U* = 45, *p* = 0.129. *n* = 13 mice (No‐Ext) and 11 mice (Ext). (D–F) Inactive‐lever responding for the mCherry and Gq groups corresponding to the acquisition‐ensemble manipulation shown in Figure 3. (D) Inactive‐lever presses during the final five FR3 acquisition sessions. Two‐way RM ANOVA: *F*_(1, 11)_ = 2.51, *p* = 0.14. (E) Inactive‐lever presses across the nine extinction sessions. Two‐way RM ANOVA: *F*_(1, 11)_ = 0.589, *p* = 0.458. (F) Inactive‐lever presses during the cue‐induced test. Unpaired *t*‐test: *t*_(11)_ = 0.92, *p* = 0.376. *n* = 7 mice (mCh) and 6 mice (Gq). (G–I) Inactive‐lever responding for the mCherry and Gq groups corresponding to the MOR‐Cre manipulation shown in Figure 4. (G) Inactive‐lever presses during the final five FR3 acquisition sessions. Two‐way RM ANOVA: *F*_(1, 16)_ = 1.31, *p* = 0.26. (H) Inactive‐lever presses across the nine extinction sessions. Two‐way RM ANOVA: *F*_(1, 16)_ = 0.226, *p* = 0.64. (I) Inactive‐lever presses during the cue‐induced test. Mann–Whitney test: *U* = 28, *p* = 0.304. *n* = 10 mice (mCh) and 8 mice (Gq).

**Figure S2:** Percentage of acutely active dMSNs during the cue‐induced test that had previously been tagged during acquisition. (A) This proportion did not differ significantly between the No‐Ext and Ext groups. Unpaired *t*‐test: *t*_(4)_ = 0.225, *p* = 0.833. Solid symbols represent animal‐level means, open symbols represent individual sections for visualization, and all statistical analyses were performed using mice as the experimental unit. *n* = 12 slices/3 mice (No‐Ext) and 14 slices/3 mice (Ext).

## Data Availability

This study did not generate new unique reagents. All data reported in this paper will be shared by the lead contact upon request. This paper does not report original code. Any additional information required to reanalyse the data reported in this paper is available from the lead contact upon request.
